# Supramolecular Networks from Block Copolymers Based on Styrene and Isoprene Using Hydrogen Bonding Motifs—Part 2: Dynamic Mechanical Analysis

**DOI:** 10.3390/ma11091688

**Published:** 2018-09-12

**Authors:** Elaine Rahmstorf, Volker Abetz

**Affiliations:** 1 Institute of Physical Chemistry, University of Hamburg, Grindelallee 117, 20146 Hamburg, Germany; elaine.rahmstorf@chemie.uni-hamburg.de; 2 Institute of Polymer Research, Helmholtz-Zentrum Geesthacht, Max-Planck-Straße 1, 21502 Geesthacht, Germany

**Keywords:** triblock copolymer, functionalization, self-complementary hydrogen bonds, supramolecular polymers, amphiphilic polymers, dynamic mechanical analysis, temperature responsiveness

## Abstract

Thermo-reversible supramolecular networks from polyisoprene-*block*-polystyrene-*block*-polyisoprene (ISI) triblock copolymers with short, functionalized polyisoprene (PI) blocks were investigated. Functional groups along the PI blocks were hydroxyl groups, ester groups with a carboxylic end-group (-O-CO-CH_2_-CH_2_-COOH), and urethane groups with an amine end-group—synthesized from various types of diamines—(-O-CO-NH-R-NH_2_). Dynamic mechanical analysis (DMA) was performed at temperatures above *T*_g_ of polystyrene (PS) to investigate the influence of the different functional groups, the molecular weight, and the composition of the triblock copolymers on the materials’ properties. Furthermore, comparisons to DMA results of diblock copolymers, modified in the same way, will be presented. Arising reversible and irreversible processes observed during DMA experiments will be compared to results from temperature-dependent Fourier transform infrared (FTIR) spectroscopy. For the elaborated systems, the transition from reversible, hydrogen-bonded to permanently cross-linked networks was observed at around 150 °C.

## 1. Introduction

Control over reversible polymer networks is becoming more and more important in developing new materials with outstanding characteristics [1,2,3,4,5]. In principle, two kinds of thermo-reversible network formation can be distinguished. On the one hand, non-covalent interactions [6,7,8,9,10,11,12,13], such as ligand-metal complexation, π-π-stacking, and hydrogen bonding, are used for the synthesis of supramolecular polymers. On the other hand, reversible covalent bonds [12,14] as used in vitrimers [2,15,16,17,18] or, for example, via a (retro-)Diels–Alder reaction [14,19,20] are investigated. Both modification approaches—covalently and non-covalently bonded—can build polymer-like arrangements consisting of lower-molecular-weight building units. The defining characteristic of such networks is their reversibility, and thus stimuli-responsive polymer properties—from elastomeric over thermoplastic to highly cross-linked—can be achieved. At room temperature, polymeric networks are present, and characteristics of high-molecular-weight polymers predominate. With increasing temperature, dissolution of the connecting groups inside the network appears, and hence fluid-like behavior dominates. Network formation occurs again during the re-cooling process.

In this work, the focus will be on thermo-reversible networks from hydrogen bonded supramolecular polymers. The modification approach to postfunctionalize the polyisoprene (PI) end-blocks of polyisoprene-*block*-polystyrene-*block*-polyisoprene (ISI) triblock copolymers and to introduce different motifs—which are able to form self-complementary hydrogen bonds—was accomplished previously (the experimental conditions and the characterization of the block copolymers are published elsewhere [21]). These structures are influenced by the type of functional groups, their arrangement, the number of groups, and their secondary interaction parameter [22,23,24] (which is affected by the spatial arrangement of hydrogen bonding donor and acceptor groups). So-called motifs consist of several hydrogen bonding donor and/or acceptor groups in one side group. Furthermore, these motifs can be divided into self-complementary and non-self-complementary groups [22]. Several multiple hydrogen bonding motifs have been investigated in the literature. Especially the work of the groups from Meijer and Stadler should be mentioned [10,25,26,27,28,29,30,31,32,33], where highly thermally responsive behavior of the investigated polymers was observed. The variation of the motif position within the polymer influences the arising architecture, such as rods, lamellae, chains, or networks. The most commonly investigated supramolecular polymers consist either of α,ω-functionalized lower-molecular-weight building blocks (forming linear structures) or of statistically functionalized polymers with higher molecular weights (building cross-linked networks).

**Figure 1:**
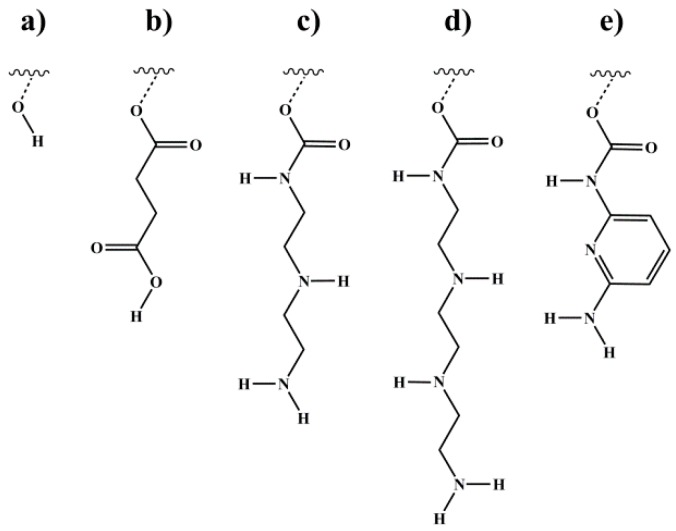
Different motifs introduced via end-block functionalization of polyisoprene (PI) double bonds. polyisoprene-*block*-polystyrene-*block*-polyisoprene (ISI) and polystyrene-*block*-polyisoprene (SI) block copolymers were modified (**a**) with 9 borabicyclo[3.3.1]nonane and subsequent oxidation to form hydroxyl groups, (**b**) via esterification with succinic anhydride (SA) to form carboxylic end-groups, and—by using 1,1′-carbonyldiimidazole as a coupling agent—with (**c**) diethylenediamine (DETA), (**d**) triethylenetriamine (TETA), and (**e**) 2,6-diaminopyridine (DAP) to form amine end-groups. Wavy lines represent the polymeric main chain. Different numbers of carbon atoms—connecting the motif and the polymeric main chain, depending on the different PI microstructures—are represented by the broken lines. Details of the syntheses are described elsewhere [21].

Regarding the presented block copolymers of this work, the concept of supramolecular polymers is combined with the approach of end-block-modified triblock copolymers from Bayer et al. [34], where new blocks were grafted on both polybutadiene end-blocks of polybutadiene-*block*-polystyrene-*block*-polybutadiene (BSB)triblock copolymers. Hence, hydrogen bonding donor and acceptor groups are introduced into the low-molecular-weight PI end-blocks of the used ISI triblock copolymers. Thus, block copolymers with already viscoelastic behavior are modified with different hydrogen bonding motifs to achieve supramolecular interactions. All presented motifs are able to form self-complementary interactions (Figure 1).

**Figure 2:**
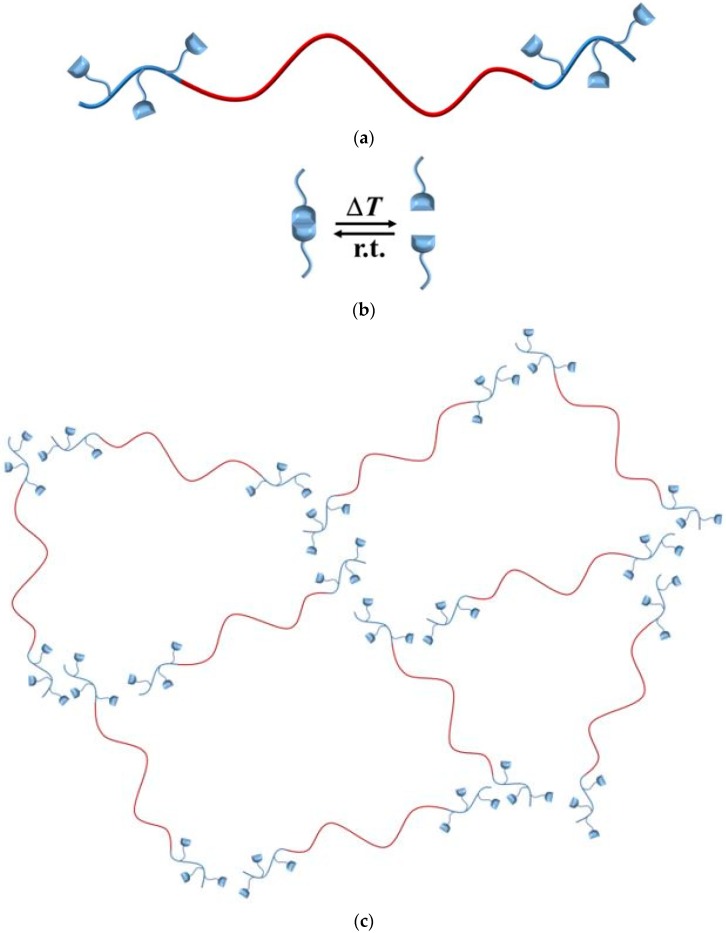
Supramolecular network formation of end-block functionalized triblock copolymers. Organization of polymer chains (**a**) is directed by hydrogen bonding motifs (**b**), and, for example, an unentangled network is formed (**c**), with polystyrene (red), and modified polyisoprene (blue).

Figure 2 shows the temperature-dependent network formation of these modified triblock copolymers, with a main PS middle block (>90 wt %) (Figure 2, red line), and small PI end-blocks (Figure 2, blue line), consisting of a few to several tens of monomeric units.

During the last decades, several approaches to investigate the influence of hydrogen bonding groups on the obtained polymer networks were accomplished. Beside infrared spectroscopy to study the temperature-dependent hydrogen bond formation [29,35], dynamic mechanical analysis is a commonly used technique to characterize these supramolecular networks [10,25,36,37,38,39].

The purpose of this work is to investigate the dynamic mechanical behavior in oscillatory shear of different modified ISI triblock copolymers and to compare it to the unfunctionalized polymers as well as to results from diblock copolymers modified in the same way.

## 2. Materials and Methods

### 2.1. Synthesis of Functionalized Block Copolymers

Several asymmetric polyisoprene-*block*-polystyrene-*block*-polyisoprene (ISI) triblock copolymers and polystyrene-*block*-polyisoprene (SI) diblock copolymers were synthesized by sequential anionic polymerization in tetrahydrofuran (THF). In this work, the double bonds of polyisoprene were modified, and several functionalization reactions were carried out to obtain different kinds of hydrogen bonds. The experimental conditions as well as the characterization of these block copolymers are published in detail elsewhere [21]. The composition and molecular weight of the originally used SI and ISI block copolymers can be found in the supplementary information (Table S1).

### 2.2. Characterization

#### 2.2.1. ^1^H NMR Spectroscopy and Calculation of Degree of Functionalization *D*_f_

To calculate the degree of functionalization *D*_f_ (in %), proton nuclear magnetic resonance (^1^H NMR) spectroscopy was applied. The spectra of all polymers were recorded on a Bruker AVANCEII (Bruker BioSpin GmbH, Karlsruhe, Germany) at 400 MHz. Tetramethylsilane (TMS) was used as an internal standard, and chloroform-d1 (CDCl_3_) or tetrahydrofuran-d8 (THF-d8) was used as a solvent. Sample concentration was 10–20 mg/mL. Measurements were recorded at 300 K. Data processing was carried out with MestReNova (Version 7.1.0, Mestrelab Research S.L., Santiago de Compostela, Spain). All spectra were normalized to the aromatic proton signal of PS (6.2–7.1 ppm). For hydroxylated polymers, *D*_f_ was calculated via the decrease of characteristic PI signals in the range of 4.4–6.1 ppm, considering the different microstructures. For the *D*_f_ calculation of the further functionalized polymers, the sum of all PI microstructure integrals (per proton) *I_H_*_,*PI*_ was set as the initial value. This value (representative for the number of PI monomer units) was assumed to be constant. For all other functionalized polymers, the integrals (per proton) *I_H_*_,*funct*._ of characteristic signals were brought into relation (Equation (1)). All calculated values of the investigated block copolymers are listed in Table 1 with regard to their modification type and the degree of functionalization *D*_f_. Related spectra and the specific signal assignments can be found in [21].

(1) $$D_{f}=\frac{I_{H,funct.}}{I_{H,PI}}\times 100\%$$

#### 2.2.2. Dynamic Mechanical Analysis

For oscillatory shear experiments, cylindrical samples with a diameter of 8 mm and a thickness of 2 mm were used. The samples were prepared by compression molding at a temperature of 135 °C for 8 min (a specimen was heated without vacuum for 3 min, under vacuum for 1 min, and finally under vacuum and with an applied force of approximately 55 kN for 4 min). A rotational rheometer (MCR 502, Anton Paar GmbH, Graz, Austria) with a plate-plate geometry in a heat chamber with nitrogen atmosphere was used. The temperature was controlled by a Peltier plate and by a constant nitrogen flow into the chamber. The gap between the lower and the upper plate was generally set to 1.0 mm. The melting time of a sample was 10 min. Before measurements, an amplitude sweep between 1% and 10% shear strain *γ* at a constant angular frequency *ω* of 10 rad/s was carried out to ensure that the chosen strain amplitude *γ*_0_ was inside the linear viscoelastic regime, so that the storage modulus *G*′ and the loss modulus *G*″ were independent of strain. In general, *γ*_0_ of 3% or 5%, depending on the investigated polymer, was chosen.

Isothermal frequency sweep experiments in oscillatory mode were performed in the linear viscoelastic regime at different temperatures (120, 140, 160, and 180 °C). A second measurement at 120 °C was performed subsequently to examine the reproducibility of the measured values. The frequency was varied between 10^2^ and 10^−2^ rad/s, starting with the lowest frequency (10^−2^ rad/s). For a better comparison of the obtained data, the Boltzmann time-temperature superposition (TTS) principle was applied to create master curves of the dynamic moduli *G*′ and *G*″ over a wide frequency range at a reference temperature of 140 °C. Temperature ramps from the glassy state at 110 °C over the molten state to 190 °C and back to 90 °C were performed at a constant angular frequency of 0.1 rad/s and a strain amplitude of 5%. The gap was kept constant during measurement. A heating and cooling rate of 1 K/min was applied. Data processing—for example, calculation of the shift factor a_T_—was performed by RheoCompass software (version 1.19.266, Anton Paar GmbH, Graz, Austria).

#### 2.2.3. Fourier Transform Infrared Spectroscopy

FTIR experiments were recorded on a Bruker FTIR Vertex 70 (Billerica, MA, USA). The measuring software was Opus 7.5. All samples were measured in the wavenumber range of 6000–400 cm^−1^ with a resolution of 2 cm^−1^ and 32 scans. For temperature-dependent FTIR experiments, samples were prepared via solution casting of a polymer film (ca. 1–2 mg in THF) coated onto a 6 mm by 1 mm potassium bromide plate. Films were dried for 16 h under vacuum at room temperature. The chosen temperature profile was similar to the temperature profile used in the oscillatory shear experiments (later referred to as “dynamic mechanical analysis (DMA) related”) and is shown in Table 2. A background measurement without a specimen in the sample holder was carried out at 30 °C and subtracted from the recorded data by the Bruker software OPUS (version 7.5, Bruker, Billerica, MA, USA). No additional data processing was implemented.

## 3. Results and Discussion

In this work, results from several ISI and SI block copolymers with molecular weights from 50–150 Da and overall PI weight fractions from 1.5–15 wt % are presented. Notation of block copolymers is written as I_x_S_y_I_z_^M^ (where x, y, and z correspond to the weight percentage of each block, and *M* indicates the number average molecular weight *M*_n_ in kg/mol [40]). For ease of comparability, polymer compositions are not recalculated after functionalization. Afterwards, the original block copolymers are extended with a motif-related suffix. Hydroxylated block copolymers are defined by the suffix –OH. The differently aminated polymers are labeled as ISI-DETA, ISI-TETA, and ISI-DAP, respectively (diethylenediamine, triethylenetetramine, and 2,6-diaminopyridine). ISI-SA indicates the carboxylated (with succinic anhydride (SA) modified) ISI. The degree of functionalization *D*_f_ defines the ratio of functionalized PI monomer units in comparison to the unfunctionalized PI, and is not related to the PS block. Calculated values of the presented block copolymers are also listed in Table 1.

### 3.1. Dynamic Mechanical Analysis

Various dynamic mechanical analysis (DMA) experiments were performed. Besides the storage and loss moduli *G*′ and *G*′′, the loss factor tan*δ* and the complex viscosity η* can be compared for the different block copolymers. For a better overview of the data, the Boltzmann time-temperature superposition (TTS) principle was used to create master curves for the angular-frequency-dependent measurements with a reference temperature *T*_ref_ of 140 °C. Entanglement molecular weights *M_e_* were calculated via the plateau modulus $G_{N}^{0}$ (Equation 2) [41,42,43], with density *ρ*, the universal gas constant *R* (8.314 J/molK), and the temperature *T* (*T*_ref_ = 413.15 K).

(2) $$G_{N}^{0}=\frac{4\rho RT}{5M_{e}}$$

To compare the calculated values, and with respect to its dominating weight fraction of at least 90 wt %, *ρ* was defined as the density of pure PS at 140 °C (1.00 g/cm³) [44,45]. $G_{N}^{0}$ is associated with the value of *G*′ at the minimum of tan*δ* [41,42]. The calculated *M_e_* of all modified and unmodified block copolymers are also listed in Table 1.

In general, amplitude sweeps before and after the measurements at one specific temperature showed no huge change in the range of values. However, it has to be considered that samples were equilibrated for 10 min in the beginning at 120 °C, where changes of the polymeric structure might occur.

#### 3.1.1. Angular-Frequency-Dependent DMA of Hydroxylated I_5_S_90_I_5_^62^ with Varying Degrees of Functionalization

**Figure 3:**
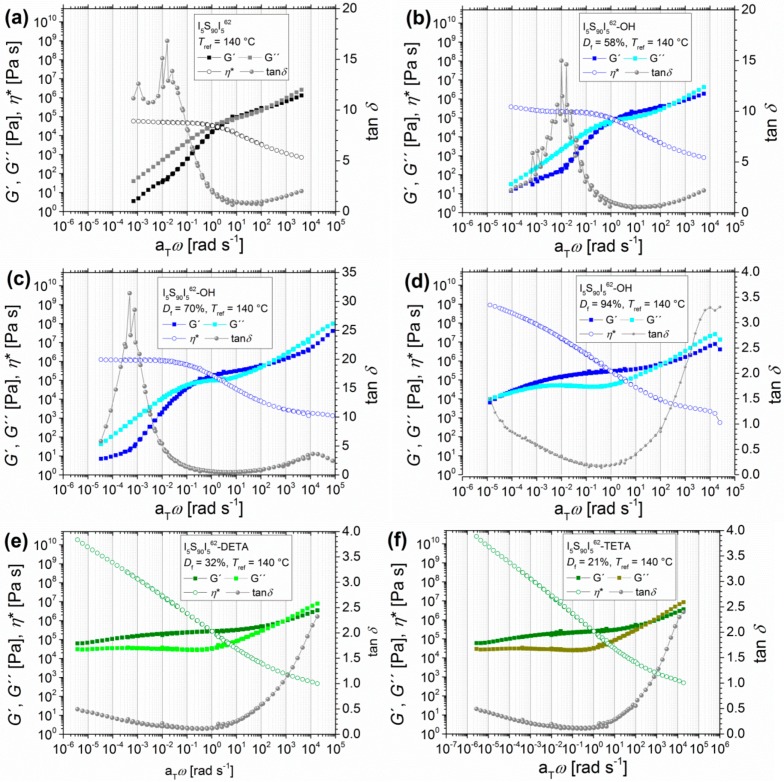

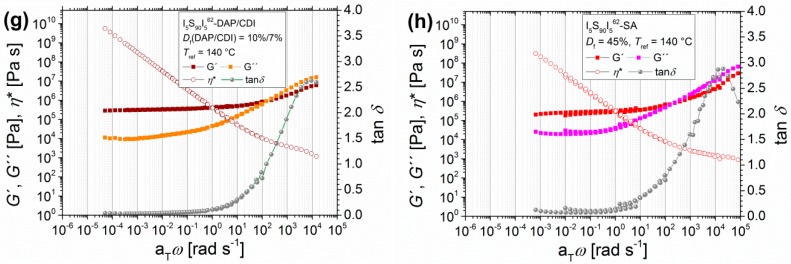
Angular-frequency-dependent master curves of I_5_S_90_I_5_^62^ before and after hydroxylation with variable degrees of functionalization *D*_f_, with the storage modulus and the loss modulus *G*′ and *G*′′, the loss factor tan*δ*, and the complex viscosity η*. Unfunctionalized polymer (**a**), hydroxylated polymer with *D*_f_ = 58% (**b**), *D*_f_ = 70% (**c**), and *D*_f_ = 94% (**d**). DETA-functionalized (**e**) with *D*_f_ = 32%, TETA-functionalized (**f**) with *D*_f_ = 21%, DAP-functionalized (**g**) with *D*_f_ = 10%, and carboxylated ISI (**h**) with *D*_f_ = 45% were synthesized from I_5_S_90_I_5_^62^-OH with *D*_f_ = 94%. Curves were measured at 120, 140, 160, and 180 °C with *T*_ref_ = 140 °C. I_5_S_90_I_5_^62^-OH (*D*_f_ = 70%) and I_5_S_90_I_5_^62^-OH (*D*_f_ = 94%) were also measured at 110 °C.

The first step of functionalization was the hydroxylation of PI double bonds. Figure 3a–d show the example of I_5_S_90_I_5_^62^ before and after hydroxylation with different degrees of functionalization (58%, 70%, and 94%). The graphs present the dynamic moduli *G*′ and *G*″ as a function of angular frequency *ω* (with the shift factor a_T_).

The transition zone can be observed for all polymers in the upper angular frequency range (with *G*′′ > *G*′). For unfunctionalized I_5_S_90_I_5_^62^, from about 2 rad/s (the low-frequency intersection *ω*_L_ of *G*′ and *G*′′) to 1 × 10^2^ rad/s (the high-frequency intersection *ω*_H_ of *G*′ and *G*′′) the entanglement plateau is located, where the elastic behavior dominates (*G*′ > *G*′′). In the lower angular frequency range, the viscous behavior dominates (*G*′′ > *G*′). Both moduli curves show a slope of 1 in this regime. In general, the cross-over *ω*_L_ of *G*′ and *G*′′ is associated with the transition from the entanglement plateau to the terminal flow zone of the polymer [46]. With regard to the complex viscosity η*, the plateau of zero viscosity *η*_0_ can be observed in the lower angular frequency range with a value of 2 × 10^6^ Pas. This behavior indicates that the polymer I_5_S_90_I_5_^62^ was not cross-linked at higher temperatures before or during the measurements [47].

After the first hydroxylation, obtaining a *D*_f_ of 58%, the *G*′ and *G*′′ functions show slopes of about 0.5. Furthermore, the complex viscosity η* again reaches a plateau with a slightly decreased zero shear viscosity *η*_0_ value of 5 × 10^5^ Pas, which is one fourth of the *η*_0_ of unmodified ISI. Apart from that, the entanglement plateau is slightly increased. The high-frequency intersection *ω*_H_ of *G*′ and *G*′′ stays constant, whereas the low-frequency intersection *ω*_L_ is shifted to lower a_T_*ω* (1 rad/s), which is an indication for the increased stiffness of the material. For the unfunctionalized polymer, the elastic part just dominates slightly over the viscous part in the rubbery plateau. With a hydroxylation *D*_f_ of 58%, the elastic part is 2 times higher than the viscous part. With further increasing of *D*_f_ to 70%, *ω*_L_ is shifted to 0.2 rad/s. The lower a_T_*ω* regime is just slightly changed, but the entanglement plateau is further enlarged, and the elastic part is now 3 times higher than the viscous part. The complex viscosity η* reaches a plateau with a zero viscosity *η*_0_ value of 1 × 10^6^ Pas. For 94% *D*_f_, the predominating viscous behavior at lower a_T_*ω* nearly disappears, and *ω*_L_ is shifted even further to 2 × 10^−5^ rad/s. Here, η* reaches no plateau. The increasing entanglement plateau shows that the polymer behaves more elastically (*G*′ > *G*′′). For unfunctionalized ISI as well as for the 58% and 70% hydroxylation products, the high value of tan*δ* at lower angular frequencies also implies that the viscous behavior dominates, which indicates the transition to the terminal flow zone. In general, the higher the tan*δ* value (with tan*δ* > 1), the more the polymers’ characteristics approach the behavior of an ideal viscous liquid, and the lower the tan*δ* value (with tan*δ* < 1), the more they approach the behavior of an ideal elastic solid.

The observed results correspond to the calculated entanglement molecular weight *M_e_*, which showed a decrease after modification (see Table 1). The *M_e_* of unfunctionalized I_5_S_90_I_5_^62^ is 21.1 kg/mol, which is in good agreement with the literature [41]. For comparison, the *M_e_* of PS is about 17 kg/mol, and that of pure PI is about 6 kg/mol [44]. With increasing hydroxylation *D*_f_, the value of *M_e_* decreases to 13.1 kg/mol (*D*_f_ = 58%), 12.5 kg/mol (*D*_f_ = 70%), and 11.4 kg/mol (*D*_f_ = 94%). This leads to the assumption that hydrogen bonds are formed, physically cross-linking the polymer, and thus the *M_e_* value is decreased and the entanglement plateau is increased. At high temperatures, when the hydrogen bonds are dissociated, the polymer again shows rather fluid-like behavior. However, the modification and so the change of the chemical structure must also be taken into account. Another important aspect is the microphase separation behavior of the block copolymers—as already mentioned in a previous publication [21]—in conjunction with the observed results from differential scanning calorimetry (DSC).

In DMA experiments, homopolymers typically show the viscoelastic behavior of a Maxwell fluid with power laws of *G*′ ~ *ω²* and *G*′′ ~ *ω* in the lower a_T_*ω* regime (with *ω* → 0). In this so-called terminal flow zone or regime, the polymer chains should be fully relaxed [46,48,49,50]. Several publications have discussed the arising power law deviation of block copolymers in the terminal regime, with dependencies for *G*′ and *G*′′ smaller than the expected 2 and 1, respectively [42,46,49,50,51]. Beside the appearance of microphase separation or superstructures, the general suppression of reptation is also mentioned. As published by Haenelt et al. [42] for a S_86_I_14_^47^ diblock copolymer, block copolymers with no or incomplete microphase separation show the beginning terminal regime, with *G*′ ~ *ω*^2^ in the beginning of the terminal regime and *G*′ ~ *ω* for the lowest angular frequencies (with *G*′′ ~ *ω* in the complete terminal regime). For microphase-separated polymers, *G*′ shows at low frequencies a plateau arising from the increased elasticity, with PI acting as a softener (with *T*_g,PI_ << *T*_g,PS_). For I_10_S_83_I_7_^67^, which was investigated by Georgopanos et al. [41]—with similar composition compared to the presented block copolymers of this work, but with different microstructures of PI—the terminal flow regime is clearly visible at a temperature of 180 °C, indicating a disordered state (at room temperature, microphase separation was observed with small angle X-ray scattering). Shabbir et al. [52] observed for poly(*n*-butyl methacrylate) similar deviations with slopes of 1 and 0.5 for *G*′ and *G*′′, respectively, which were explained by the presence of hydrogen bonds. Analogous values were observed for diblock copolymers with miscible blocks [53,54].

In this work, for I_5_S_90_I_5_^62^ before and after hydroxylation up to 70%, power laws of *G*′ ~ *ω*^0.5–1^ and *G*′′ ~ *ω* are observed for the lowest angular frequencies, which can be indicated as the beginning of the terminal regime or a rather nearly terminal behavior, even though no homopolymer-like behavior with full polymer chain relaxation is present. A molecular weight with a multiple of the entanglement molecular weight could lead to suppression of polymer chain reptation, and thus the terminal regime is shifted to even lower angular frequencies. To verify, if the terminal flow zone—for example, for 94% hydroxylation—is completely missing or just shifted to higher temperatures, further measurements at higher temperatures (for example 200 °C) would be beneficial. For the utilized devices in this work, only measurements up to 190 °C were possible. To demonstrate, if the unmodified block copolymers are partially microphase-separated, small angle x-ray scattering (SAXS) experiments are crucial. As presented in a previous publication [21], microphase separation became apparent after modification of PI blocks. Unfunctionalized block copolymers as well as modified block copolymers with very small end-blocks have so far revealed no structure formation (films were prepared by solvent annealing as well as by melt pressing).

#### 3.1.2. Angular-Frequency-Dependent DMA of Different Modified I_5_S_90_I_5_^62^ and I_3_S_94_I_3_^117^

For further modification with different functional groups, first the polymer I_5_S_90_I_5_^62^ with a hydroxylation *D*_f_ of 94% is presented to outline the difference between unfunctionalized, hydroxylated, aminated, and carboxylated polymers. The resulting master curves of oscillatory shear experiments are also shown in Figure 3. It must be taken into consideration that none of the presented modifications reached the former 94% *D*_f_ of hydroxylated I_5_S_90_I_5_^62^.

These polymers indeed are showing interesting rheological properties. At higher a_T_*ω*, all polymers show the beginning of the transition zone (*G*′′ > *G*′), similar to what was already seen in the previous section. However, at lower a_T_*ω* for DETA-, TETA-, DAP-, and SA-functionalized ISI, the entanglement plateau changes significantly. While I_5_S_90_I_5_^62^ and I_5_S_90_I_5_^62^-OH reach the transition to the terminal flow zone at higher temperatures, all further functionalized I_5_S_90_I_5_^62^ stay in the entanglement plateau with *G*′′ < *G*′. For DETA- and TETA-functionalized ISI, the convergence of *G*′ and *G*′′ at very low a_T_*ω* can be observed. A direct comparison shows a minimal difference in the values of *G*′ and *G*′′; both are negligibly lower for I_5_S_90_I_5_^62^-TETA. Again, the question arises as to whether higher temperatures would lead to a terminal flow behavior, and thus to the domination of the viscous behavior. Unfortunately, temperatures higher than 190 °C cannot be implemented with the experimental setup used in this work. For the DAP- and SA-functionalized polymers, the transition to the terminal flow zone disappears completely. *G*′ and *G*′′ are roughly constant at lower a_T_*ω* and show frequency-independent behavior, which is characteristic of highly cross-linked polymers [47,55].

A similar effect on the entanglement plateau after introduction of hydrogen bonding motifs was observed by Shabbir et al. [52] for poly(*n*-butyl methacrylate) with different degrees of carboxylation. With increasing *D*_f_, the entanglement plateau also increased, and the terminal regime disappeared. No chemical cross-linking was observed due to the lower experimental temperatures of less than 50 °C. In that case, physical cross-linking, initiated by hydrogen bonds, was certainly observed. For the I_5_S_90_I_5_^62^-SA it has to be further investigated whether this behavior is related to chemical cross-linking (permanent) or to physical cross-linking (reversible); for example, due to hydrogen bonding (see discussion Section 3.3.). Therefore, additional temperature-dependent infrared spectroscopy was performed to investigate the reversibility of hydrogen bonds inside the polymer (see Section 3.2.1.).

The master curves of tan*δ* confirm that functionalized polymers show cross-linking behavior at lower frequencies. Unfunctionalized and hydroxylated polymers show the highest tan*δ* values for lower angular frequencies, which indicates fluid-like behavior. In contrast, the DETA- and TETA-functionalized polymers show much lower tan*δ* values at low a_T_*ω*. I_5_S_90_I_5_^62^-SA and I_5_S_90_I_5_^62^-DAP even show the lowest, and also constant, tan*δ* values in this a_T_*ω* range, which indicates solid-like behavior and thus cross-linking. The calculated entanglement molecular weights *M_e_* show again a decrease after functionalization. For I_5_S_90_I_5_^62^, the *M_e_* is 21.1 kg/mol, and hydroxylated I_5_S_90_I_5_^62^ (*D*_f_ = 94%) shows an *M_e_* of 11.4 kg/mol. SA- and TETA-functionalized I_5_S_90_I_5_^62^ show an *M_e_* of about 12 kg/mol, whereas the *M_e_* of DETA- and DAP-functionalized I_5_S_90_I_5_^62^ were calculated to be about 9 kg/mol. Hence, *M_e_* is approximately halved.

**Figure 4:**
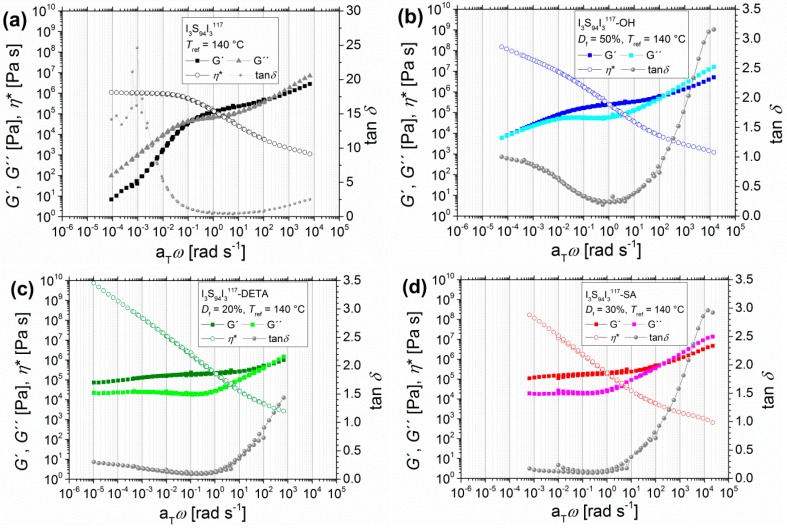
Master curves of angular-frequency-dependent measurements of I_3_S_94_I_3_^117^ with different functionalities, with the storage modulus and the loss modulus *G*′ and *G*′′, the loss factor tan*δ*, and the complex viscosity η*. (**a**) Unfunctionalized; (**b**) hydroxylated (*D*_f_ = 50%); (**c**) DETA-functionalized (*D*_f_ = 20%); and (**d**) SA-functionalized (*D*_f_ = 30%). Curves were measured at 120, 140, 160, and 180 °C with *T*_ref_ = 140 °C.

With increasing molecular weight of the triblock copolymer, the same tendency of change in mechanical behavior can be observed. Figure 4 shows the modified triblock copolymer I_3_S_94_I_3_^117^ with about twice the molecular weight of the first example from Figure 3, but with lower PI content and a lower degree of functionalization.

The transition zone (*G*′′ > *G*′) of all polymers can be observed in the upper angular frequency range. For I_3_S_94_I_3_^117^, the entanglement plateau (*G′* > *G*′′) can be found from about 0.2–200 rad/s. In comparison to I_5_S_90_I_5_^62^, the intersection *ω*_L_ is shifted from 2 rad/s for I_5_S_90_I_5_^62^ to 0.2 rad/s for I_3_S_94_I_3_^117^ due to the higher molecular weight [47]. In the lower angular frequency range, the viscous behavior dominates (*G*′′ > *G*′). However, the storage modulus *G*′ again shows a deviation from the slope 2, and consequently the terminal zone is not finally reached. The *η*_0_ reaches a constant value of 1 × 10^6^ Pas. In accordance with I_5_S_90_I_5_^62^, power laws of *G*′ ~ *ω* and *G*′′ ~ *ω* are again observed. This behavior indicates also that the polymer I_3_S_94_I_3_^117^ was not cross-linked at higher temperatures before or during oscillatory shear measurements up to 180 °C. For the hydroxylated DETA- and SA-functionalized I_3_S_94_I_3_^117^, the same changes as for I_5_S_90_I_5_^62^ were observed, and therefore will not be discussed further in detail. Calculation of *M_e_* showed results differing from and less pronounced than those from I_5_S_90_I_5_^62^. For I_3_S_94_I_3_^117^ and I_3_S_94_I_3_^117^-SA, the *M_e_* was calculated to be ~15 kg/mol, I_3_S_94_I_3_^117^-OH showed an *M_e_* of 11.0 kg/mol, and that of I_3_S_94_I_3_^117^-DETA was 13.1 kg/mol. These values indicate that with increasing molecular weight of the PS middle block, the *M_e_* of ISI is more similar to that of pure PS (17 kg/mol [44]) and modification of PI has less influence on *M_e_*.

**Figure 5:**
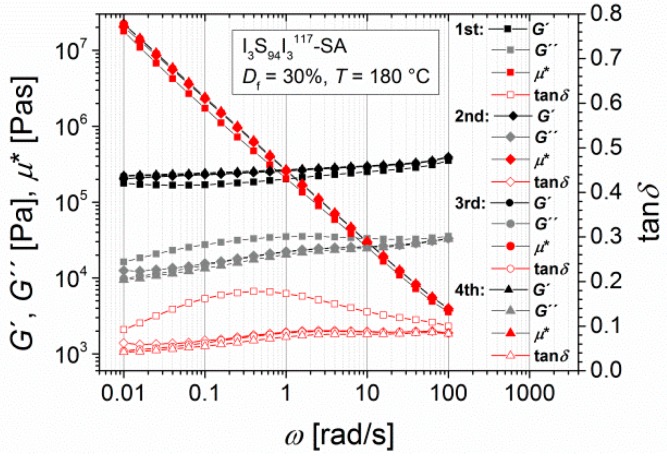
Multiple angular-frequency-dependent measurements of I_3_S_94_I_3_^117^-SA (*D*_f_ = 30%), with the storage modulus and the loss modulus *G*′ and *G*′′, the loss factor tan*δ*, and the complex viscosity η*. Curves were measured at 180 °C from 100 to 0.1 rad/s (1st and 3rd experiment) and from 0.1 to 100 rad/s (2nd and 4th experiment) with a strain amplitude of 5%.

Multiple measurements of I_3_S_94_I_3_^117^-SA at 180 °C (Figure 5) show the long-term influence of high temperatures. The main change in mechanical characteristics results during the first frequency sweep, which corresponds to a time scale of 1 h. During the second, third, and fourth frequency sweeps, no further changes were observed. Hence, the resulting mechanical properties were attained in a predictable time slot.

#### 3.1.3. Lowering the PI Contents in ISI and Its Influence on the Results from DMA

**Figure 6:**
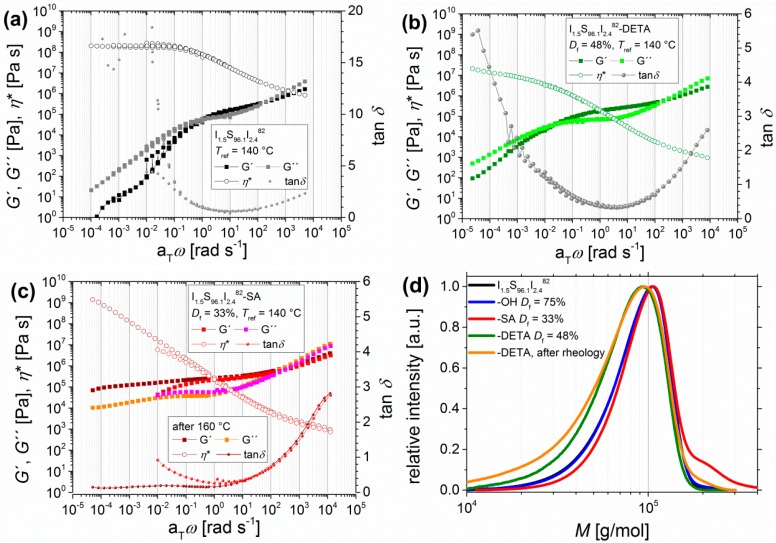
Master curves of angular-frequency-dependent measurements of I_1.5_S_96.1_I_2.4_^82^ (**a**) after DETA-functionalization (**b**) with *D*_f_ = 48%, and after carboxylation (**c**) with *D*_f_ = 33%. Curves were measured at 120, 140, 160, and 180 °C with *T*_ref_ = 140 °C, with the storage modulus and the loss modulus *G*′ and *G*′′, the loss factor tan*δ*, and the complex viscosity η*. For I_1.5_S_96.1_I_2.4_^82^-SA, two master curves were created: before cross-linking (using the measurements at 120 and 140 °C), indicated in red/pink, and after cross-linking (using the measurements at 160, 180, and 120 °C), indicated in brown/orange. (**d**) shows the size exclusion chromatography (SEC) curves of I_1.5_S_96.1_I_2.4_^82^ (black) after hydroxylation (*D*_f_ = 75%, blue), after DETA-functionalization (*D*_f_ = 48%, green), and after carboxylation (*D*_f_ = 33%, red) and of the I_1.5_S_96.1_I_2.4_^82^-DETA after DMA (brown). I_1.5_S_96.1_I_2.4_^82^-SA was not soluble after DMA.

For some of the investigated polymers, a change in mechanical behavior during the oscillatory shear experiment was observed. At 160 °C, during the measurement, a stronger increase and the domination of the elastic part were detected. For data evaluation, two master curves could be calculated: one for 120 and 140 °C and another one after reaching 160 °C. To investigate the influence of the functional groups, their absolute number was indirectly decreased by lowering the PI content from 6 wt % to 4 wt %. Figure 6 shows the resulting master curves from I_1.5_S_96.1_I_2.4_^82^.

With I_1.5_S_96.1_I_2.4_^82^-SA, it has been observed that even for very low PI contents with less than 5 wt % the DMA curve still shows cross-linking behavior at temperatures higher than 140 °C (Figure 6c, brown and orange squares), whereas the I_1.5_S_96.1_I_2.4_^82^-DETA shows nearly a terminal flow zone at high temperatures. For very low PI contents, the cross-linking effect of I_1.5_S_96.1_I_2.4_^82^-SA does not occur at temperatures lower than 140 °C (Figure 6c, red and pink). At low a_T_*ω*, even the convergence of *G*′ and *G*′′ can be seen, which indicates the dissolution of hydrogen bonds, and so the increasingly liquid-like behavior of the polymer. After the oscillatory shear experiment, I_1.5_S_96.1_I_2.4_^82^-SA is insoluble, whereas I_1.5_S_96.1_I_2.4_^82^-DETA is still soluble after the experiment, and so SEC (Figure 6d, orange) was performed. No or just a slight change of molecular weight distribution—for example, due to temperature-induced decomposition—was observed, and consequently no chemical cross-linking occurred. Hence, it can be concluded that up to 140 °C the entanglement plateau of ISI-SA is enlarged because of hydrogen bonds before the thermo-induced cross-linking process begins. I_1.5_S_96.1_I_2.4_^82^-DETA, on the other hand, shows thermo-reversible characteristics up to 180 °C.

#### 3.1.4. Comparison of Functionalized ISI Triblock Copolymers to OH- and SA-functionalized SI Diblock Copolymers

**Figure 7:**
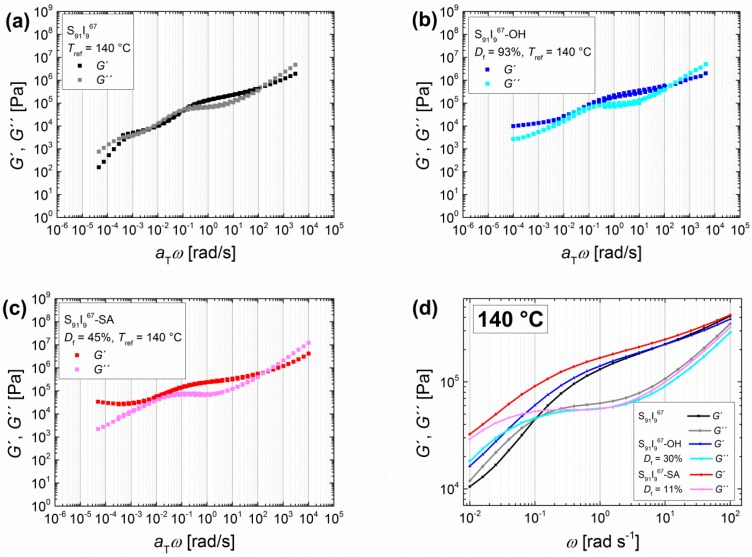
Angular-frequency-dependent master curves of polystyrene-*block*-polyisoprene with different functionalities, with the storage modulus and the loss modulus *G*′ and *G*′′. Unfunctionalized S_91_I_9_^67^ (**a**) followed by hydroxylated (**b**, *D*_f_ = 93%) and carboxylated (**c**, *D*_f_ = 45%) polymer. Curves were measured at 120, 140, 160, and 180 °C with *T*_ref_ = 140 °C. (**d**) shows the same original polymer with different *D*_f_ after hydroxylation (*D*_f_ = 30%) and carboxylation (*D*_f_ = 11%). Curves were measured at 140 °C.

Beside the modification type, the architecture of the block copolymer could also influence the resulting properties. Therefore, SI diblock copolymers were hydroxylated and carboxylated with different *D*_f_. The following master curves (Figure 7) were obtained from unfunctionalized, hydroxylated (*D*_f_ = 93%), and carboxylated (*D*_f_ = 45%) S_91_I_9_^67^. Furthermore, measurements at 140 °C of the same original diblock copolymer S_91_I_9_^67^, but with lower *D*_f_ after hydroxylation (*D*_f_ = 30%) and carboxylation (*D*_f_ = 11%), are shown.

The transition zone is again observed for all polymers. At lower a_T_*ω*, the entanglement plateau increases for S_91_I_9_^67^-OH and S_91_I_9_^67^-SA, and the crossing point *ω*_L_ of *G*′ and *G*′′ is shifted to lower a_T_*ω*. Whereas unfunctionalized SI reaches for lower a_T_*ω* the beginning of the terminal flow zone, both functionalized SI stay in the entanglement plateau. Also here, the frequency-independent behavior of *G*′ and *G*′′ implies a strong cross-linking [47]. In comparison to the observations of Haenelt et al. [42,56], the diblock copolymers in this work with a lower PI content of 9 wt %, but with introduced polar groups, show curves that are comparable to microphase-separated SI with higher PI contents of more than 20 wt %. Finally, in the entanglement plateau, similar changes as for triblock copolymers can be observed, but compared to I_5_S_90_I_5_^62^—with equivalent molecular weight and degree of functionalization—the impact of modification on the diblock copolymer is less pronounced.

#### 3.1.5. Temperature-Dependent Dynamic Mechanical Analysis

**Figure 8:**
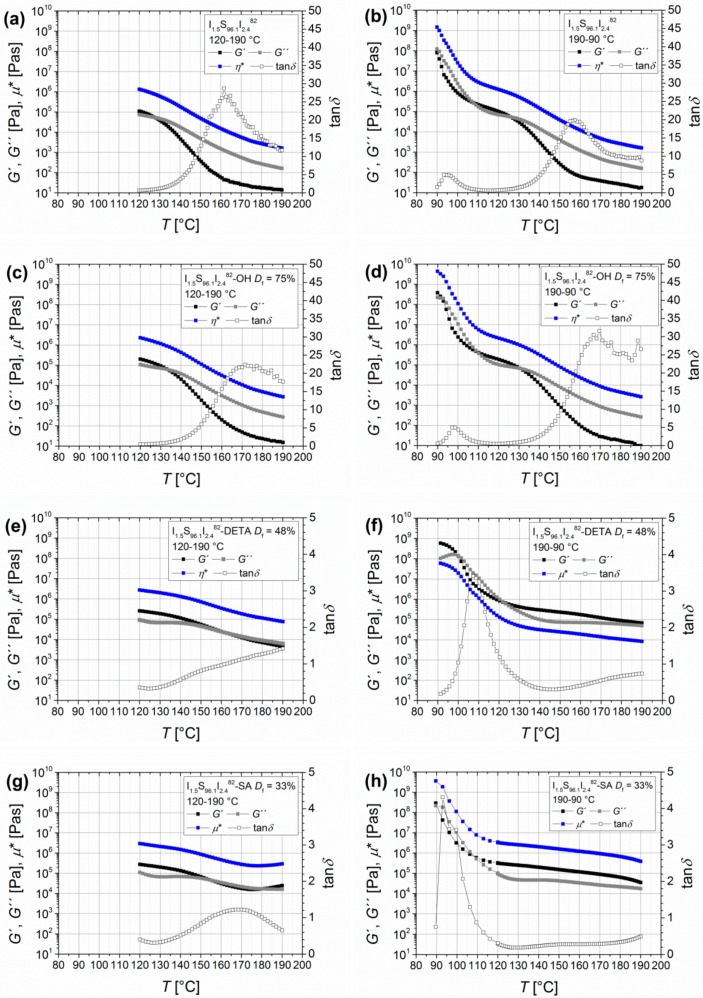
Temperature-dependent oscillatory shear curves of I_1.5_S_96.1_I_2.4_^82^ (**a**,**b**), I_1.5_S_96.1_I_2.4_^82^-OH with *D*_f_ = 75% (**c**,**d**), I_1.5_S_96.1_I_2.4_^82^-DETA with *D*_f_ = 48% (**e**,**f**), and I_1.5_S_96.1_I_2.4_^82^-SA with *D*_f_ = 33% (**g**,**h**). (**a**,**c**,**e**,**g**) show the heating curves (120–190 °C) and (**b**,**d**,**f**,**h**) show the cooling curves (190–90 °C) for each polymer. Storage modulus *G*′ (black), loss modulus *G*′′ (grey), complex viscosity η* (blue), and loss factor tan*δ* (open rectangles) were measured with a constant angular frequency of 0.1 rad/s and a strain amplitude of 5%.

For comparison of temperature-dependent oscillatory shear experiments, I_1.5_S_96.1_I_2.4_^82^ with different functionalities was chosen. Figure 8 shows both the heating (left side, 120–190 °C) and the cooling (right side, 190–90 °C) process. The graphs present the dynamic moduli *G*′ and *G*″ as a function of temperature *T*.

For the unfunctionalized and hydroxylated I_1.5_S_96.1_I_2.4_^82^, a reversible, temperature-dependent behavior was observed. The heating as well as the cooling are congruent. At 128 °C (I_1.5_S_96.1_I_2.4_^82^) and at 130 °C (I_1.5_S_96.1_I_2.4_^82^-OH), the crossing of *G*′ and *G*′′ can be seen, and with further heating the viscous behavior of the polymer dominates. The phenomenon of microphase separation for the unfunctionalized polymer, as determined by Georgopanos et al. [41] for ISI with larger PI contents of 17 wt %, and indicated by a second plateau with *G*′ > *G*′′, was not observed. The DETA- and SA-functionalized polymers show slightly different behavior of heating and cooling curves. The crossing of *G*′ and *G*′′ is shifted to higher temperatures, which is probably caused by hydrogen bonds. For I_1.5_S_96.1_I_2.4_^82^-DETA, this crossing appears at around 160 °C, and for I_1.5_S_96.1_I_2.4_^82^-SA at around 152 °C. Whereas the response of I_1.5_S_96.1_I_2.4_^82^-DETA is interestingly changing after reaching 190 °C, I_1.5_S_96.1_I_2.4_^82^-SA already shows a change in the *G*′/*G*′′ ratio during the heating phase at around 180 °C. For I_1.5_S_96.1_I_2.4_^82^-SA, this indicates that an irreversible process is happening during the heating phase. Multiple measurements showed that these changes of the *G*′ and *G*′′ curves only occurred during the first heating interval. Afterwards, the heating and cooling curves were congruent.

### 3.2. Fourier Transform Infrared Spectroscopy (FTIR)

Infrared spectroscopy was used to investigate the temperature-dependent behavior in order to confirm the observed behavior in oscillatory shear experiments (hereinafter referred to as “DMA-related” FTIR spectroscopy). FTIR spectra of the complete wavenumber range are available in the supplementary information (Figures S1 and S2).

Related to the previous publication [21], in this work, the main focus will be on the carbonyl region [57,58], where complexed and free species can be distinguished. Furthermore, the main emphasis is on SA- and DETA-functionalized ISI. When *T*_g_ is exceeded (at *T* > 110 °C), the polymer perhaps starts to creep, and the thickness changes. For some samples, the baseline of higher wavenumbers is shifted partially to lower absorption values. It was not always observed, and not polymer-dependent. Therefore, it should not be related to changes of the absorption coefficient, for example because of changes in the chemical structure due to thermal-induced reactions.

#### DMA-Related Temperature Profile of I_1.5_S_96.1_I_2.4_^82^-DETA and of I_1.5_S_96.1_I_2.4_^82^-SA

**Figure 9:**
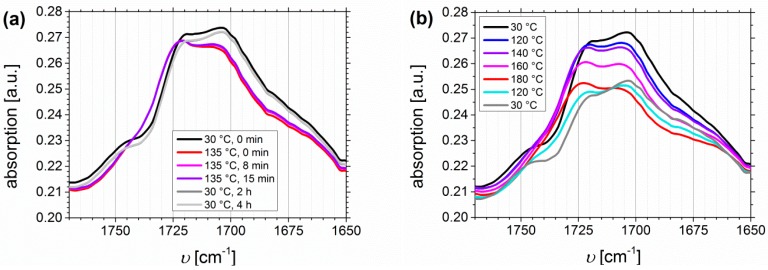

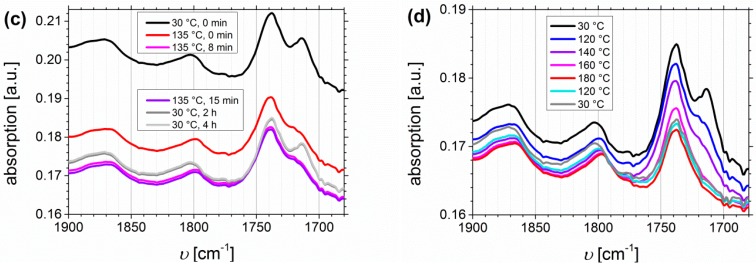
Temperature-dependent FTIR spectra of I_1.5_S_96.1_I_2.4_^82^-DETA with *D*_f_ = 48% (**a**,**b**), and of I_1.5_S_96.1_I_2.4_^82^-SA with *D*_f_ = 33% (**c**,**d**) with a “DMA-related” temperature profile: (**a**,**c**) related to melt pressing and (**b**,**d**) related to the oscillatory shear experiment with 1 h controlled holding of each temperature.

In this DMA-related approach, polymer films were heated using a temperature profile similar to the one used for the DMA measurements (for the temperature profile see Table 2). To identify the temperature of complete hydrogen bond dissociation, the maximum change of the ratio between complexed and free species of the carbonyl band can be used. Figure 9 shows the carbonyl region of I_1.5_S_96.1_I_2.4_^82^-DETA (*D*_f_ = 48%) and I_1.5_S_96.1_I_2.4_^82^-SA (*D*_f_ = 33%). The free and complexed carbonyl absorption bands of ISI-DETA (Figure 9a,b) are located at 1721 and 1705 cm^−1^, respectively. The complexed carboxyl and the ester carbonyl bands of ISI-SA (Figure 9c,d) are located at 1736 and 1714 cm^−1^, respectively. Further signals from the carbonyl groups are not observed.

During the melt-pressing step (Figure 9a,c), thermo-reversible behavior of both functionalization types can be observed. The ratio of complexed and free species before and after this process is not changed. It confirms the observations from the oscillatory shear experiments, where irreversible mechanical changes occur at temperatures higher than 140 °C. Also with time (up to 4 h at 30 °C), no further change of signals—for example, the further increase of the complexed carbonyl band—can be observed. A temperature profile that was predominant during the oscillatory shear experiments (Figure 9b,d; “DMA-related”) revealed a significant change in the carbonyl region for both types of functionalization. The complexed carbonyl band is decreasing with heating, and thus the ratio of complexed to free carbonyl bands is changing. I_1.5_S_96.1_I_2.4_^82^-DETA showed a major change already at 120–160 °C, even though the band at 1705 cm^−1^ is further decreasing from 160 to 180 °C. After cooling to 120 °C, this decrease is not reversible, even though the ratio of free to complexed carbonyl bands is recovered at 30 °C. This indicates that hydrogen bond formation still takes place, but with a lower number of hydrogen bonds inside the polymer. For I_1.5_S_96.1_I_2.4_^82^-SA, the maximum change for the ester/complexed-carbonyl ratio can be seen at 160 °C, where the band at 1714 cm^−1^ disappears. The significant absorption decrease from the band located at 1736 cm^−1^ starts at around 160 °C. After cooling to 30 °C, neither this decrease nor the hydrogen bond formation itself is reversible. Comparing these observations to bands from PS—where the most characteristic vibration is the out-of-plane mode of the monosubstituted aromatic group located at ~700 cm^−1^ [59] (which is not overlaying with other signals, and so can be used as an internal reference)—the intensity change of the band at 700 cm^−1^ is negligible, and it confirms that only a change of the introduced hydrogen bonding motif takes place.

Since this specific polymer/motif arrangement has not been described in the literature so far, investigations of similar chemical structures are considered in the following. Different explanations are possible. Velada et al. [60] observed three degradation products for different poly(mono-*n*-alkyl itaconates), including water, carbon dioxide, and the corresponding alkyl alcohol, and concluded deesterification, anhydride formation, and decarboxylation reactions had occurred. As the predominant process, from 120 to 160 °C the cyclic anhydride products caused signals to occur at 1860 and 1780 cm^−1^. In general, anhydride formation would also lead to cross-linking. For poly(mono-*n*-alkyl itaconates), a decarboxylation process was observed at *T* > 180 °C [60]. Pure poly(lactic acid) [61,62] begins to decompose at ~275 °C, which is observable with the C–O stretching at 1260 and 1100 cm^−1^. For I_1.5_S_96.1_I_2.4_^82^-SA, this region remains unchanged. Also, for poly(lactic acid), MacNeill et al. [63] observed thermal degradation under vacuum at *T* > 230 °C, with the anhydride formation as the main degradation process. Matsuzaki et al. [64] concluded that for poly(*tert*-butyl methacrylate), a two-step reaction took place: first a deesterification followed by subsequent anhydride formation. After annealing at 170 °C and 200 °C, respectively, strong absorption bands assigned to the anhydride group were observed at 1022, 1760, and 1810 cm^−l^. Unfortunately, for the polymers of this work, these anhydride signals would overlap with the unchanged ester carbonyl group as well as with the CH out-of-plane deformation vibrations of the PS phenyl group [65] at 1945, 1875, 1802, 1745, and 1675 cm^−1^. Summarizing, it seems reasonable to suppose that more than one degradation process took place during the DMA-related experiments.

### 3.3. Discussion on Cross-Linking Behavior during Heat Treatment

Beside the conclusions from the FTIR and DMA measurements so far, one additional observation was made for all carboxyl- and most of the amine-functionalized polymers (except I_1.5_S_96.1_I_2.4_^82^-DETA). They became insoluble after heat treatment. For each DMA and FTIR experiment (and also for films from solvent casting annealed at 120 °C), after the addition of solvent, polymers were swollen but not solved. Different solvents and solvent mixtures with regard to their solubility parameters were tested. One explanation can be found in the structure formation during solvent evaporation or during the cooling process. In both cases—in the dissolved state or in the molten state—the polymer chains can move freely, and both functionalized polymer end-blocks can arrange inside the PS matrix. This arrangement is enhanced by the hydrogen bonds of the modified end-blocks. When the solution is concentrated or the polymer melt is cooled, the mobility of PS chains is decreased. Below *T*_g_, this formed network shows strongly cross-linked behavior, and the solvation process is more difficult to complete. Beside this physical cross-linking, chemical cross-linking could occur as well. For example, the remaining peroxides from the used THF could enhance the cross-linking of the remaining double bonds inside the PI block. Certainly, no cross-linking was observed for unfunctionalized or hydroxylated polymers. ^1^H NMR studies before and after DMA showed no decrease of PI double-bond signals. A possible reason for chemical cross-linking is also the presence of oxygen. First, it should be pointed out that all oscillatory shear measurements were performed under a nitrogen atmosphere. Stability tests of pure PI were performed under nitrogen and under air by Haenelt et al. [42], where slight increases of *G*′ and *G*′′ appeared under nitrogen, but they were explained by changes in morphology. Furthermore, oxygen could be dissolved by the amine or carboxylic side groups due to their polar character, which could also enhance the formation of radicals during heat treatment, and thus lead to cross-linking of the remaining PI double bonds.

**Figure 10:**
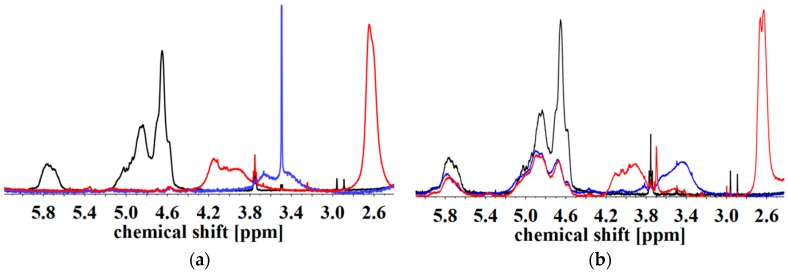
^1^H NMR spectra of I_5_S_90_I_5_^62^-SA with *D*_f_ = 45% (**a**) and I_3_S_94_I_3_^117^-SA with *D*_f_ = 30% (**b**). Unfunctionalized (black), hydroxylated (blue), and with SA carboxylated (red) in CDCl_3_. Spectra were normalized to aromatic protons of PS (6.2–7.2 ppm, 5H).

However, Figure 10 shows the ^1^H NMR spectra of two ISI-SA with different *D*_f_ and different amounts of remaining PI double bonds. The absolute number of functionalized monomer units is approximately similar (45 for I_3_S_94_I_3_^117^-SA and 38 for I_5_S_90_I_5_^62^-SA). The only difference of both block copolymers is the content of remaining double bonds. I_5_S_90_I_5_^62^-SA has no apparent double bonds (Figure 10a), whereas I_3_S_94_I_3_^117^-SA has about 50% (Figure 10b).

For both polymers, a quite similar entanglement plateau is reached (Figure 3 and Figure 4) independent from the number of remaining PI double bonds. Thus, the remaining double bonds do not influence the resulting curves, which confirms the anhydride formation process as the explanation for cross-linking. In general, a closer FTIR analysis of PI-related bands, and their possible decrease because of radical cross-linking, is not promising due to their low signal intensity in FTIR spectra.

**Figure 11:**
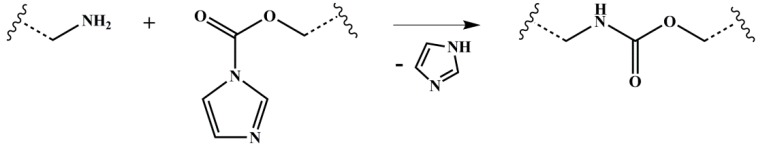
Reaction scheme for both intra- and intermolecular cross-linking reactions of amine- and CDI-functionalized polymers under imidazole elimination (CDI: 1,1′-carbonyldiimidazole). Wavy lines represent the polymeric main chain. Different numbers of atoms connecting the functional group and the polymeric main chain (depending on the different PI microstructures and the motif) are represented by the broken lines.

For DAP- or DETA-functionalized polymers with the remaining CDI groups, the reaction shown in Figure 11 is also possible.

Arguments against this hypothesis can be found in the direct comparison between DETA- and DAP-functionalized polymers (Figure 3). Both master curves show similar cross-linking behavior, for example, also independent from the molecular weight of the original polymer (Figure 4). Having regard to the ^1^H NMR spectra published earlier [21], for ISI-DETA (Figure S3a), no remaining CDI-characteristic signals can be observed at 7.4 and 8.1 ppm, indicating a full conversion to the DETA-functionalized polymer. For DAP-functionalized polymers (Figure S3b), the CDI-characteristic signals are present, but negligible. Hence, the reaction of Figure 11 should not occur. One additional observation for S_85_I_15_^51^-DAP is the decomposition of the DAP motif during the synthesis. This can be seen in Figure S3b, where the *D*_f_ decreases at 100 °C from 18% (after a reaction time of 7 h) to 12% (after 3 days) and to 4% (after 4 days). However, finally this decomposition should not lead to cross-linking. Despite that, a direct addition of amine-groups to remaining double bonds (hydroamination) is not possible without previous activation of the C=C double bond [66]. The behavior of completely dried I_5_S_90_I_5_^62^-DETA (at room temperature under vacuum) after addition of THF confirms the presence of strong hydrogen bonds, since the dissolving process took a long time, and was not complete, which could explain the cross-linked-like characteristics.

With I_1.5_S_96.1_I_2.4_^82^ (Figure 8), a new point of view comes up. With very low numbers of functional groups, I_1.5_S_96.1_I_2.4_^82^-DETA shows nearly terminal flow behavior at high temperatures. Here, no irreversible cross-linking can be observed, the sample is soluble after the DMA measurement, and the SEC curves show no or marginal change of the molecular weight distribution. I_1.5_S_96.1_I_2.4_^82^-SA, on the other hand, again shows not even the transition to the terminal flow region at low angular frequencies, but stays in the rubbery plateau. This confirms the explanation by means of the thermally induced cross-linking by the carboxyl group as discussed in the previous section.

## 4. Conclusions

The end-block functionalization of polyisoprene-*block*-polystyrene-*block*-polyisoprene triblock copolymers leads to significant changes in mechanical behavior. Strong networks were accomplished after introduction of different hydrogen bonding motifs into the PI block. Dependencies on the type of motif, the degree of functionalization, and the absolute number of functional groups were observed. DMA results for temperatures higher than the *T*_g_ of the main PS block (>100 °C) were presented. Even at 120 °C, when a relatively high percentage of hydrogen bonds should be dissociated, an increase in the rubbery plateau was achieved for all modified polymers due to the introduced hydrogen bonding motifs. Each motif had a different effect on the resulting curves of storage and loss moduli *G*′ and *G*′′, respectively. The lowest influence on the mechanical characteristics was observed after the introduction of the hydroxyl groups, due to the weakest ability to form hydrogen bonds, even though also the variation of the degree of hydroxylation showed significant changes of the rubbery plateau. After modification of triblock copolymers (with PI contents of 5−10 wt %) with SA, DETA, DAP, and TETA, quite similar value ranges of *G*′ and *G*′′ were reached depending on the original polymer. However, small differences were observed for higher temperatures and lower angular frequencies, respectively. Whereas ISI-SA and ISI-DAP showed quite constant and thus angular-frequency-independent values (indicating a chemical cross-linking), the curves of *G*′ and *G*′′ showed for ISI-DETA and ISI-TETA a convergence. With a decreasing PI fraction to less than 5 wt % (as presented with I_1.5_S_96.1_I_2.4_^82^), ISI-SA still confirmed an irreversible, permanent cross-linking behavior, whereas ISI-DETA reached the transition to the terminal flow zone (with domination of the viscous-like behavior), and thus confirmed a reversible, temperature-dependent cross-linking. The DMA results of functionalized polystyrene-*block*-polyisoprene diblock copolymers showed a reduced impact of the introduced motifs on the mechanical properties in contrast to the end-block-functionalized triblock copolymers.

The DMA-related FTIR experiments—with the main focus on SA- and DETA-functionalized triblock copolymers—confirmed for both modification types the thermo-reversible behavior up to 135 °C. At higher temperatures up to 180 °C, irreversible changes in the structure were detected. For DETA-functionalized triblock copolymers, the decomposition of the motif seemed to occur, but the remaining motifs were still able to form hydrogen bonds. On the contrary, SA-functionalized triblock copolymers showed decomposition with disappearance of the carboxyl-end-group characteristic IR bands and the loss of the ability to form hydrogen bonds, which lead to the assumption of anhydride formation.

In general, the properties of systems—such as the presented I_1.5_S_96.1_I_2.4_^82^-DETA—change at high temperatures from that of a high-molecular-weight network to that of single, weakly entangled polymer chains compared to the extremely low viscous behavior of the low-molecular-weight building units normally used for supramolecular polymers. This might be an advantage if homogenous composites of polymer and nanoparticles have to be processed. Compared to α,ω-functionalized polymers, the higher number of hydrogen bonding motifs of end-block-functionalized triblock copolymers lead to stronger networks due to a higher number of connecting groups inside the crosslinking “points” or microdomains of the network and due to a higher probability of successful interacting groups. Summarizing, the observed thermo-reversible interactions confirmed the polymers’ applicability as a thermo-responsive material to create materials with temperature-dependent characteristics, such as, for example, an easier processability—in consequence of lower viscosities at high temperatures—compared to permanently cross-linked networks. Nonetheless, the thermally induced cross-linking of ISI-SA—which in the presented results was reached in less than one hour—can also be utilizable for permanently cross-linked networks if desired. Thus, both types of networks should be advantageous to prepare tunable material characteristics.

## Figures and Tables

**Table 1:** Measured and calculated data of different functionalized and unfunctionalized ISI and SI. Listed are the polymeric precursor, the introduced functional group FG_int_, the degree of functionalization *D*_f_, the polydispersity index *Đ*, the onset of glass transition temperature *T*_g_, and the entanglement molecular weight *M_e_*. Data of original block copolymers are listed in Table S1. A part of the data was taken from a preceding publication [21].

Polymer	FG_int_	*Đ*	*D*_f_ (%)	*T*_g_ (°C) *^a^*	*M_e_* (kg/mol)
I_5_S_90_I_5_^62^	-	1.3	-	86.0	21.1
I_5_S_90_I_5_^62^	OH	1.2	58	93.2	13.1
I_5_S_90_I_5_^62^	OH	1.3	70	*^c^*	12.5
I_5_S_90_I_5_^62^	OH	1.3	94	101.5	11.4
I_5_S_90_I_5_^62^	DETA	*^b^*	32	97.8	8.9
I_5_S_90_I_5_^62^	TETA	1.4	21	94.8	11.9
I_5_S_90_I_5_^62^	DAP/CDI	1.3	10/7	100.7	9.2
I_5_S_90_I_5_^62^	SA	2.0	45	97.3	12.2
I_1.5_S_96.1_I_2.4_^82^	-	1.2	-	95.0	17.3
I_1.5_S_96.1_I_2.4_^82^	OH	1.2	75	97.9	*^c^*
I_1.5_S_96.1_I_2.4_^82^	DETA	1.3	48	98.9	12.1
I_1.5_S_96.1_I_2.4_^82^	SA	1.2	33	100	11.1
I_3_S_94_I_3_^117^	-	1.2	-	83.7	15.3
I_3_S_94_I_3_^117^	OH	1.5	50	101.1	11.0
I_3_S_94_I_3_^117^	DETA	2.3	20	98.2	13.1
I_3_S_94_I_3_^117^	SA	1.7	30	99.8	16.2
S_91_I_9_^67^	-		-	98.2	16.2
S_91_I_9_^67^	OH	1.2	30	99.1	16.0
S_91_I_9_^67^	SA	1.4	11	100.8	15.1
S_91_I_9_^67^	OH	1.1	92	97.8	9.0
S_91_I_9_^67^	SA	1.5	45	99.0	11.3

*^a^* Onset; *^b^* not analyzed (due to insolubility); *^c^* not measured.

**Table 2:** Measuring profile of temperature-dependent, oscillatory-shear-experiment-like FTIR spectroscopy (later referred to as “dynamic mechanical analysis (DMA) related”). Numbers represent the waiting time in minutes after reaching a certain temperature (1st), and after the last measurement (2nd–4th). Up to four measurements were taken at each temperature.

*T* (°C)	1st (min)	2nd (min)	3rd (min)	4th (min)
30	0			
135	0	7	8	
30	0	120	120	
120	0	10	30	30
140	0	10	30	30
160	0	10	30	30
180	0	10	30	30
120	0	10	30	30
30	0	120	120	

## Supplementary Materials

The following are available online at http://www.mdpi.com/1996-1944/11/9/1688/s1, Table S1: Investigated di- and triblock copolymers and the dispersity indices of precursor *Đ*_Pre_ as well as *Đ*_poly_ of the resulting SI or ISI block copolymers. All *Đ* were determined from SEC measurements. PI-Precursors were not measured due to their too-low molecular weight. The degree of polymerization *P*_n_ is given for the polystyrene (S) and polyisoprene (I) monomer units, Figure S1: Full-range, temperature-dependent FTIR spectra of I_1.5_S_96.1_I_2.4_^82^-DETA with *D*_f_ = 48% with a “DMA-related” temperature profile: (a) related to melt pressing, and (b) related to an oscillatory shear experiment with 1 h controlled holding of temperature, Figure S2: Full-range, temperature-dependent FTIR spectra of I_1.5_S_96.1_I_2.4_^82^-SA with *D*_f_ = 33% with a “DMA-related” temperature profile: (a) related to melt pressing, and (b) related to an oscillatory shear experiment with 1 h controlled holding of temperature, Figure S3: (a) ^1^H NMR spectra of S_85_I_15_^51^ (black), after hydroxylation (blue), and after reaction with CDI (orange) and DETA (green) in CDCl_3_. (b) ^1^H NMR spectra of CDI-functionalized S_85_I_15_^51^ (top), and after addition of DAP with reaction times of 7 h, 3 days, and 4 days (from top to the bottom) in CDCl_3_. ^1^H NMR spectra were normalized to aromatic protons of PS (6.2–7.2 ppm, 5H). (Spectra were published before [21]).
