# Supplementary material for: Supramolecular Networks from Block Copolymers Based on Styrene and Isoprene Using Hydrogen Bonding Motifs—Part 2: Dynamic Mechanical Analysis
Source: Materials (Basel). 2018 Sep 12;11(9):1688. doi: 10.3390/ma11091688 (PMC6164313; doi:10.3390/ma11091688)
Supplement: Supplementary file 1 [file materials-11-01688-s001.pdf]

materials

Supplementary Information

Supramolecular Networks from Block Copolymers Based on Styrene and Isoprene Using Hydrogen Bonding Motifs—Part 2: Dynamic Mechanical Analysis

Elaine Rahmstorf [1] and Volker Abetz [1,2,*]

[1] Institute of Physical Chemistry, University of Hamburg, Grindelallee 117, 20146 Hamburg, Germany; elaine.rahmstorf@chemie.uni-hamburg.de
[2] Institute of Polymer Research, Helmholtz-Zentrum Geesthacht, Max-Planck-Straße 1, 21502 Geesthacht, Germany; volker.abetz@hzg.de
* Correspondence: volker.abetz@hzg.de; Tel.: +49-40-42838-3460

Table S1: Di- and triblock copolymers and the dispersity indices of precursor $Đ_{Pre}$ as well as $Đ_{poly}$ of the resulting SI or ISI block copolymers. All $Đ$ were determined from SEC measurements. PI-Precursors were not measured due to too low molecular weight. The degree of polymerization P_n is given for the polystyrene (S) and polyisoprene (I) monomer units. Data were published before [21].

Polymer	$Đ_{S\text{-}Pre}$	$Đ_{SI}$	P_n (S/I)	Polymer	$Đ_{IS\text{-}Pre}$	$Đ_{ISI}$	P_n (I/S/I)
$S_{91}I_{9}^{67}$	1.1	1.1	585/89	$I_{5}S_{90}I_{5}^{62}$	1.2	1.3	46/536/46
$S_{85}I_{15}^{51}$	1.4	1.4	416/112	$I_{3}S_{94}I_{3}^{117}$	1.2	1.2	52/1056/52
$S_{41}I_{59}^{31}$	1.1	1.1	123/273	$I_{1.5}S_{96.1}I_{2.4}^{82}$	1.2	1.2	18/757/29
$S_{51}I_{49}^{5}$	1.3	1.1	24/35	$I_{0.6}S_{98.8}I_{0.6}^{98}$	1.2	1.2	9/935/9
				$I_{0.7}S_{98.5}I_{0.8}^{149}$	1.2	1.4	10/932/11

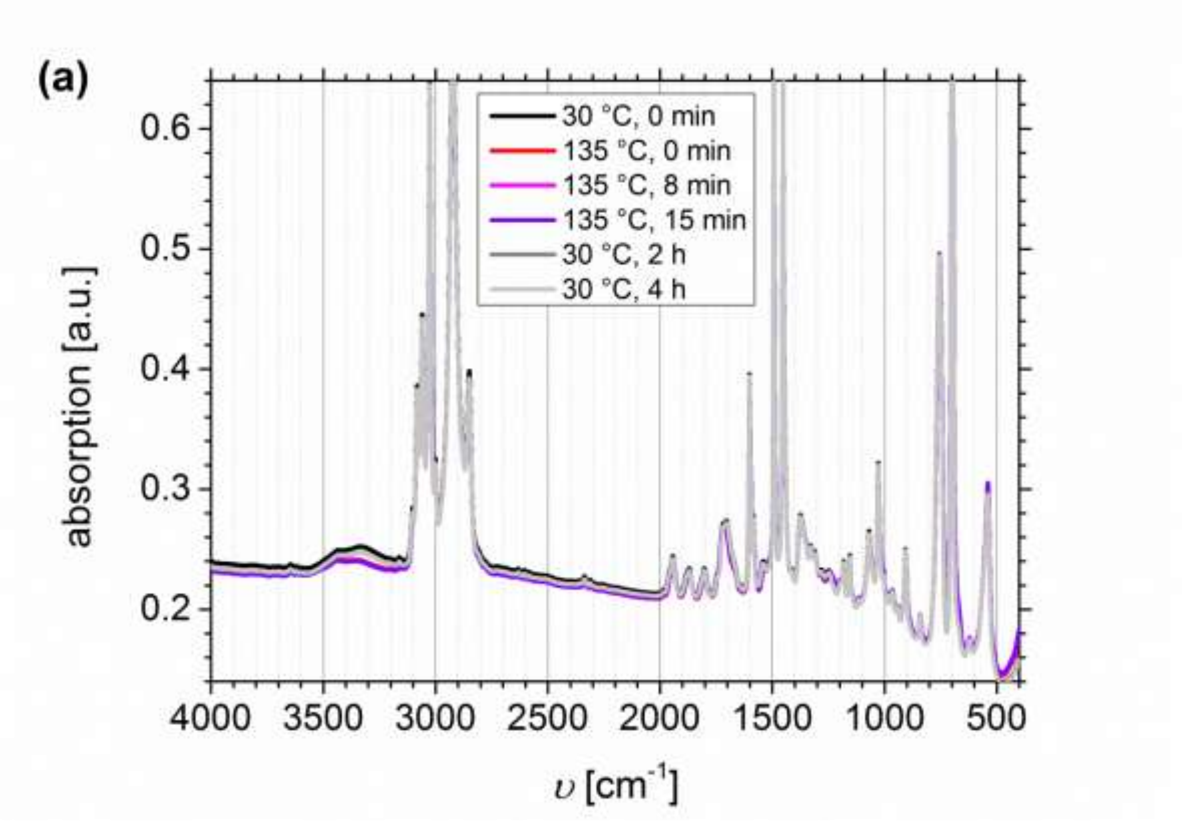

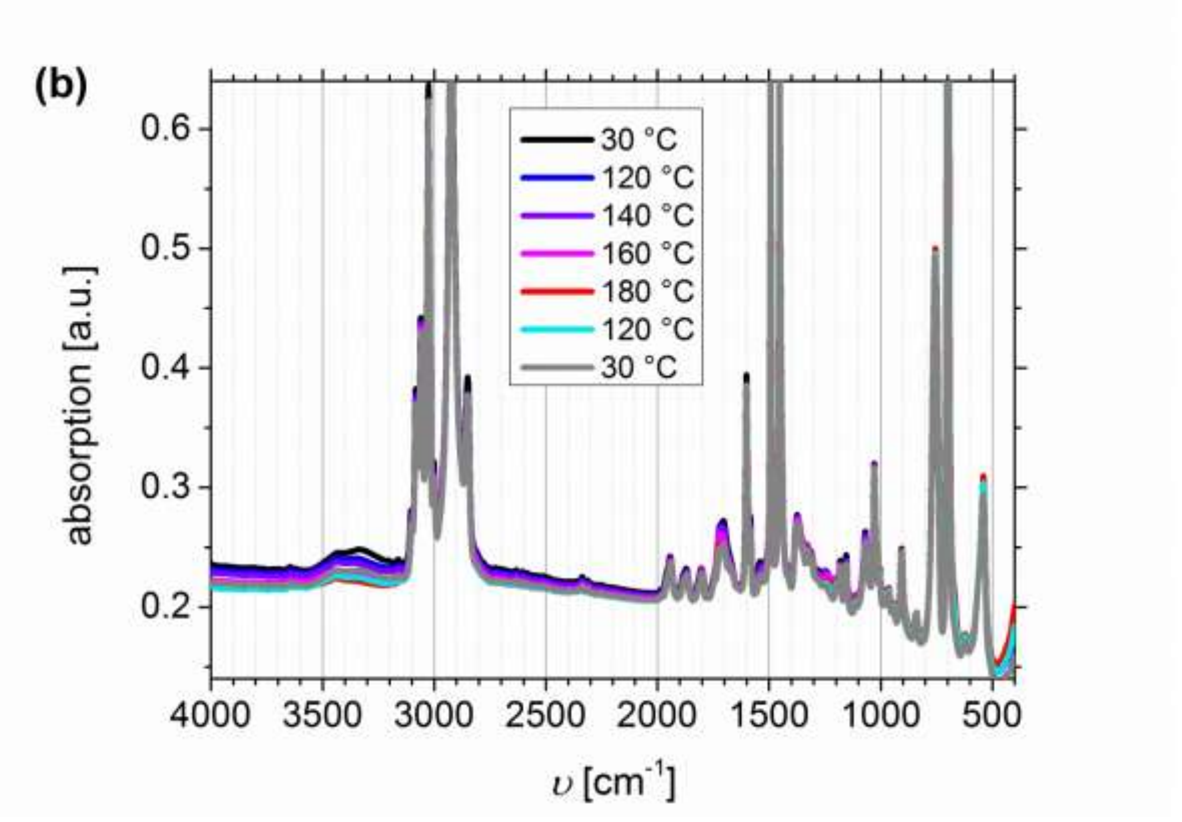

Figure S1: Full range temperature dependent FTIR spectra of $I_{1.5}S_{96.1}I_{2.4}{}^{82}$-DETA with D_f = 48% with a "DMA related" temperature profile: (**a**) related to melt pressing, and (**b**) related to oscillatory shear experiment with 1 h controlled holding of temperature.

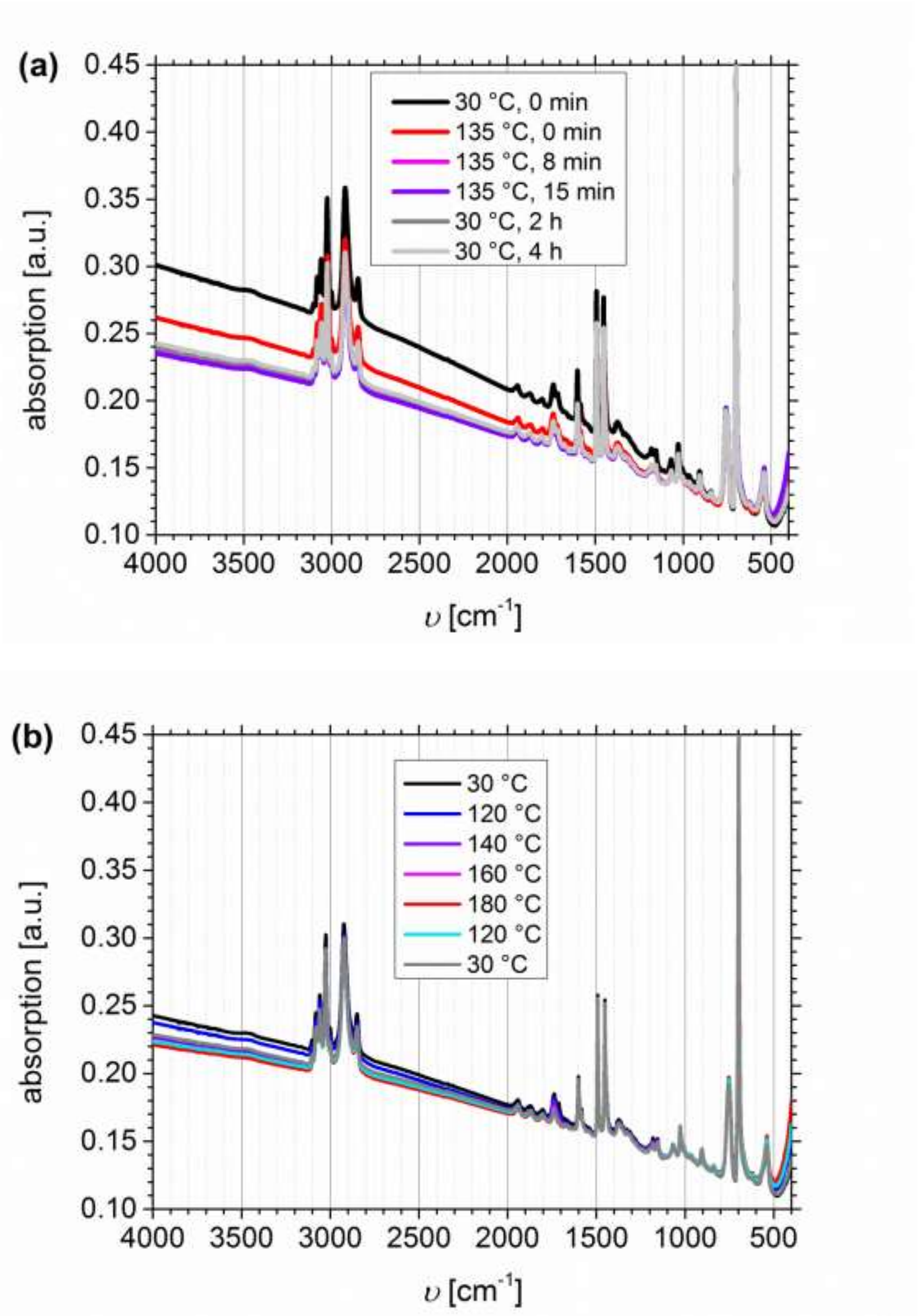

Figure S2: Full range temperature dependent FTIR spectra of $I_{1.5}S_{96.1}I_{2.4}^{82}$-SA with D_f = 33% with a "DMA related" temperature profile: (**a**) related to melt pressing, and (**b**) related to oscillatory shear experiment with 1 h controlled holding of temperature.

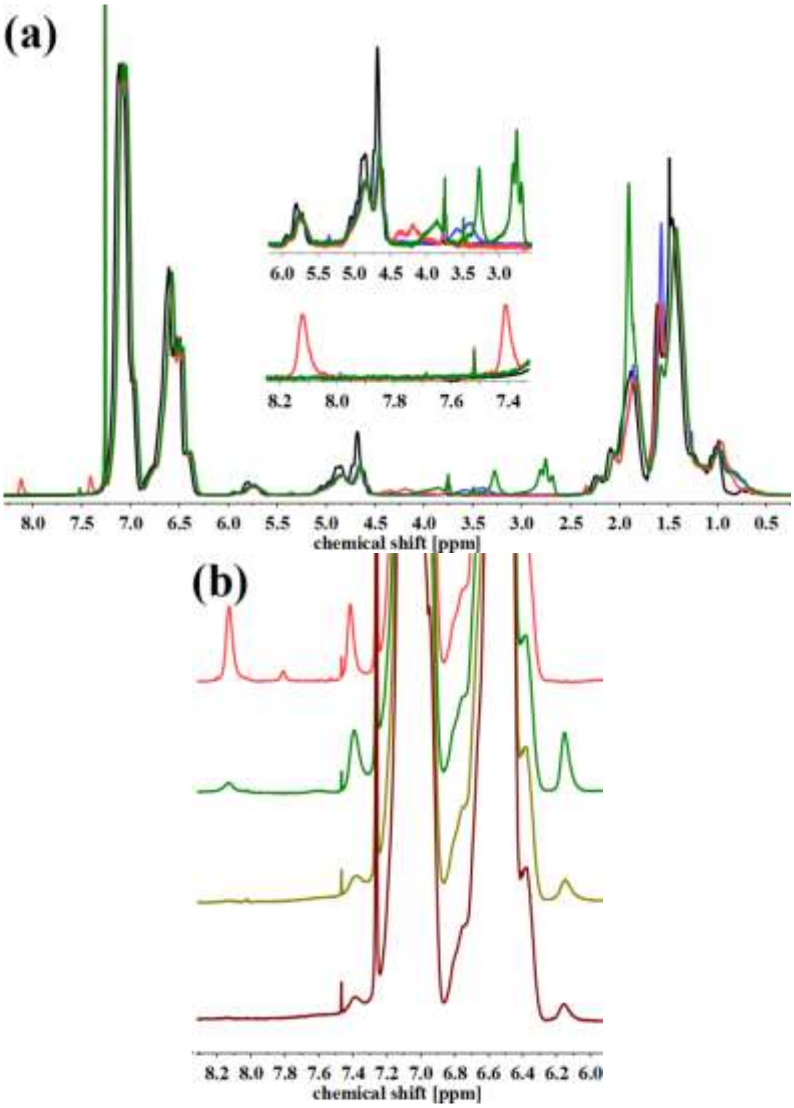

Figure S3: (**a**) ^{1}H NMR spectra of $S_{85}I_{15}^{51}$ (black), after hydroxylation (blue), and after reaction with CDI (orange) and DETA (green) in $CDCl_3$. (**b**) ^{1}H NMR spectra of CDI-functionalized $S_{85}I_{15}^{51}$ (top), and after addition of DAP with reaction times of 7 h, 3 d and 4 d (from top to the bottom) in $CDCl_3$. ^{1}H NMR spectra were normalized to aromatic protons of PS (6.2–7.2 ppm, 5H). (Spectra were published before [21])

Reference (according to the reference number of the article)

21. Rahmstorf, E.; Abetz, V. Supramolecular Networks from Styrene and Isoprene Block Copolymers Based on Hydrogen Bonding Motifs—Part 1: Synthesis and Characterization. *Materials* **2018**, *11*(9), 1608, doi:10.3390/ma11091608.
